# Endogenous Prostaglandins and Afferent Sensory Nerves in Gastroprotective Effect of Hydrogen Sulfide against Stress-Induced Gastric Lesions

**DOI:** 10.1371/journal.pone.0118972

**Published:** 2015-03-16

**Authors:** Marcin Magierowski, Katarzyna Jasnos, Slawomir Kwiecien, Danuta Drozdowicz, Marcin Surmiak, Malgorzata Strzalka, Agata Ptak-Belowska, John L. Wallace, Tomasz Brzozowski

**Affiliations:** 1 Department of Physiology, Jagiellonian University Medical College, Cracow, Poland; 2 Department of Physiology & Pharmacology, University of Calgary, Calgary, Canada; University of Missouri, UNITED STATES

## Abstract

Hydrogen sulfide (H_2_S) plays an important role in human physiology, exerting vasodilatory, neuromodulatory and anti-inflammatory effects. H_2_S has been implicated in the mechanism of gastrointestinal integrity but whether this gaseous mediator can affect hemorrhagic lesions induced by stress has been little elucidated. We studied the effect of the H_2_S precursor L-cysteine, H_2_S-donor NaHS, the H_2_S synthesizing enzyme (CSE) activity inhibitor- D,L-propargylglycine (PAG) and the gastric H_2_S production by CSE/CBS/3-MST activity in water immersion and restraint stress (WRS) ulcerogenesis and the accompanying changes in gastric blood flow (GBF). The role of endogenous prostaglandins (PGs) and sensory afferent nerves releasing calcitonin gene-related peptide (CGRP) in the mechanism of gastroprotection induced by H_2_S was examined in capsaicin-denervated rats and those pretreated with capsazepine to inhibit activity of vanilloid receptors (VR-1). Rats were pretreated with vehicle, NaHS, the donor of H_2_S and or L-cysteine, the H_2_S precursor, with or without the concurrent treatment with 1) nonselective (indomethacin) and selective cyclooxygenase (COX)-1 (SC-560) or COX-2 (rofecoxib) inhibitors. The expression of mRNA and protein for COX-1 and COX-2 were analyzed in gastric mucosa pretreated with NaHS with or without PAG. Both NaHS and L-cysteine dose-dependently attenuated severity of WRS-induced gastric lesions and significantly increased GBF. These effects were significantly reduced by pretreatment with PAG and capsaicin denervation. NaHS increased gastric H_2_S production via CSE/CBS but not 3-MST activity. Inhibition of COX-1 and COX-2 activity significantly diminished NaHS- and L-cysteine-induced protection and hyperemia. NaHS increased expression of COX-1, COX-2 mRNAs and proteins and raised CGRP mRNA expression. These effects of NaHS on COX-1 and COX-2 protein contents were reversed by PAG and capsaicin denervation. We conclude that H_2_S exerts gastroprotection against WRS-induced gastric lesions by the mechanism involving enhancement in gastric microcirculation mediated by endogenous PGs, sensory afferent nerves releasing CGRP and the activation of VR-1 receptors.

## Introduction

Hydrogen sulfide (H_2_S) is a gaseous mediator, which plays an important role in human physiology [1]. Like other endogenous gasotransmitters, nitric oxide (NO) and carbon monoxide (CO), H_2_S can modulate vascular tone [2, 3]. H_2_S is mostly generated *via* L-cysteine metabolism and the activity of two pyridoxal-5`-phosphate (P5P, vitamin B_6_) dependent enzymes: cystathionine β-synthase (CBS) and cystathionine γ-lyase (CSE) [4, 5]. However, this molecule may be synthesized by another pathway, mainly within mitochondria, that involves the activity of 3-mercaptopyruvate sulfotransferase (3-MST) and cysteine aminotransferase [6].

H_2_S can evoke anti-inflammatory and pro-inflammatory effects depending on lower and higher concentration, respectively [7, 8]. The vasodilatory effects of the H_2_S donor, sodium hydrosulfide (NaHS) in blood vessels was confirmed by Kubo *et al*. [9] and Zhao *et al*. [10] who demonstrated that this donor results in a direct vasorelaxation of vascular smooth muscle acting as KATP channel opener.

Morsy *et al*. [11] demonstrated that treatment with H_2_S donors reduced acetaminophen-induced hepatotoxicity in mice, which was correlated with a decreased expression of tumor necrosis factor-α (TNF-α) and enhanced expression of cyclooxygenase (COX)-2. H_2_S may play an important role within the gastrointestinal (GI) tract [12] because Wallace *et al*. [13] observed that treatment with L-cysteine and H_2_S donors accelerated the healing of experimental chronic gastric ulcers. Additionally, endogenous synthesis of H_2_S is significantly increased in the colon of animals with experimental colitis, compared to healthy colonic tissue [14, 15].

However, relatively little is known concerning the contribution of H_2_S to the mechanism of gastroprotection against acute gastric lesions, in particular, those induced by stress. Therefore, we attempted to determine the effect of pretreatment with NaHS and L-cysteine against gastric lesions induced by water immersion and restraint stress (WRS) and accompanying changes in the gastric blood flow (GBF) and the gastric mucosal production of H_2_S assessed by CSE/CBS/3-MTS activity. We examined the mechanism of the potential protective action of H_2_S released from NaHS or that generated from its precursor L-cysteine against stress ulcerogenesis with special reference to endogenous prostaglandins (PGs) and sensory afferent nerves releasing calcitonin gene-related peptide (CGRP) by using animals with non-selectively and selectively inhibited COX-1 and COX-2 activity and those with functionally ablated sensory neurons by capsaicin, respectively [16, 17]. Finally, the expression of proinflammatory cytokine TNF-α and its plasma levels as well as the mucosal expression of mRNAs for CGRP, COX-1 and COX-2 and the COX-1 and COX-2 protein concentration were analyzed in rats treated with NaHS with or without capsaicin denervation in order to determine the relationship between PG and sensory nerves releasing vasodilatatory mediators.

## Materials and Methods

### Animals & Ethics Statement

Male Wistar rats with an average weight of approximately 250 g were fasted for 24 h, with free access to water before being exposed to 3.5 h of WRS. The Ethics Committee for Animal Research of Jagiellonian University Medical College approved all procedures, and experiments were run according to the principles of Helsinki Declaration.

### Chemicals and drugs application, determination of the number of lesions and gastric blood flow

Animals were selected into the groups pretreated with: A) vehicle (saline; 1 ml/rat), B) L-cysteine (2–80 mg/kg i.g.) and C) NaHS (0.1–5 mg/kg i.g.) with or without the combination with D, L-propargylglycine (PAG 30 mg/kg i.g.), an inhibitor of CSE activity. All chemicals were of the highest purity grade and were purchased from Sigma-Aldrich, Schnelldorf, Germany. In a separate groups of rats, NaHS and L-cysteine were administered with or without the co-treatment with 16,16 dmPGE_2_ or with the non-selective COX inhibitor—indomethacin (5 mg/kg i.p., Sigma-Aldrich, Schnelldorf, Germany) or selective to COX-1 inhibitor—SC-560 (5 mg/kg i.p., Cayman Chemical, Ann Arbor, USA) and COX-2 inhibitor—rofecoxib (30 mg/kg i.g., Pfizer, Illertissen, Germany) [16, 17]. In rats of series D, capsazepine (10 mg/kg i.g., Sigma-Aldrich, Schnelldorf, Germany) was administered in order to inhibit vanilloid receptor (VR-1) according to the evidence published previously [18, 19].

In rats of series E, capsaicin (Sigma-Aldrich, Schnelldorf, Germany) was administered in a large dose of 125 mg/kg s.c. to induce the functional ablation of sensory nerves as reported in our previous studies [20]. Briefly, 2 weeks before the experiment capsaicin was administered subcutaneously in three doses 25, 50 and 50 mg/kg (total dose: 125 mg/kg). To check the effectiveness of the capsaicin denervation, a drop of 0.1 mg/ml solution of capsaicin was instilled into the eye of each rat and the protective wiping movement was counted [21, 22].

All COX-1 and COX-2 inhibitors, PGE_2_ and capsazepine were administered 30 min prior to the subsequent application of NaHS or L-cysteine, followed 30 min later by 3.5 h of WRS. For this purpose rats were immobilized in individual Bolman’s cages and immersed in the water (22°C) to the level of the xyphoid cartilage as described in our previous studies [20]. At the termination of WRS animals were anesthetized with ketamine (10 mg/kg i.p.) and their abdomen was opened and the stomach was exposed for the GBF measurement by H_2_-gas clearance technique as described in detail previously [16, 19, 20]. The GBF was measured in fundic part of the gastric mucosa not involving mucosal lesions. Average values of three measurements were determined and expressed as a percentage change of the value determined in intact gastric mucosa. Gastric lesions number was determined with computerized planimetry (Morphomat, Carl Zeiss, Berlin, Germany) as described before [16, 23]. The blood samples from *vena cava* and gastric tissue were collected and stored in −80°C for further biochemical and molecular analysis.

### H_2_S production in gastric mucosa determined by CSE/CBS/3-MST activity

The ability of gastric mucosa to produce H_2_S *via* CSE/CBS or 3-MST pathway was measured in homogenized tissue in the presence of exogenous substrates using a previously described zinc (Zn)-trapping assay [13, 15]. Briefly, gastric mucosa was quickly isolated, snap-frozen, and stored at −80°C. The gastric tissue was homogenized in ice-cold 50 mM potassium phosphate buffer, pH 8.0 (12% w/v). The homogenate (0.5 ml) and buffer (433 μl) were then cooled on ice for 10 min before L-cysteine (10 mM) and P5P (2 mM) or α-ketoglutarate (α-KG; 100 μM) were added (up to 1 ml of total volume). A smaller 1,5-ml tube containing a piece of filter paper (0.5×1.5 cm) soaked with zinc acetate (1%; 0.3 ml) was put inside the larger vial. The vials were then flushed with nitrogen gas for 20 s and capped with an airtight serum cap. The vials were then incubated in a shaking water bath at 37°C for 90 min. Trichloroacetic acid (TCA; 50%; 0.5 ml) was then injected into the reaction mixture through the serum cap. The mixture was left to stand for another 60 min in 50°C to allow for the trapping of evolved H_2_S by the Zn acetate. The serum cap was then removed and N, N-dimethyl-p-phenylenediamine sulfate (20 mM; 50 μl) in 7.2 M HCl and FeCl_3_ (30 mM; 50 μl) in 1.2 M HCl were added to the inner tube. After 20 min, absorbance at 670 nm was measured with a microplate reader (Biotek Instruments, ELx808, VT, USA). The calibration curve of absorbance *vs*. H_2_S concentration was obtained by using NaHS solution of varying concentrations. Addition of the substrate, L-cysteine (10 mM), was necessary for detection of H_2_S synthesis. H_2_S biosynthesis *via* CSE and CBS required the presence of P5P (2 mM unless otherwise stated), while that *via* 3-MST required α-KG.

### Expression of mRNA for COX-1, COX-2, TNF-α and CGRP in the rat gastric mucosa determined by reverse transcriptase-polymerase chain reaction (RT-PCR)

Biopsy samples of gastric mucosa weighing about 200 mg were scraped off from oxyntic mucosa using a slide glass and immediately snap frozen in liquid nitrogen, and stored at −80°C until analysis. The total RNA was extracted from the mucosal samples by a guanidium isothiocyanate/phenol chloroform method using a kit from Stratagene (Heidelberg, Germany) according to methods described by Chomczynski and Sacchi [24]. The concentration of RNA in RNase-free Tris EDTA buffer was measured at absorption of 260 nm wavelengths by spectrophotometry.

Five μg of total cellular RNA single-stranded cDNA was generated using StrataScript reverse transcriptase and oligo(dT) primers (Stratagene). The polymerase chain reaction mixture was amplified in a DNA thermal cycler (Perkin-Elmer-Cetus, Norwalk, CT). The nucleotide sequences of the primers used in PCR are presented in *Table 1*.

**Table 1:** Sense and antisense primers used in the assessment of mRNA expression for β-actin, CGRP, COX-1, COX-2 and TNF-α by reverse transcriptase polymerase chain reaction (RT-PCR).

GENE	PRIMER
β-actin	Sense: 5’-TTG TAA CCA ACT GGG ACG ATA TGG-3’Antisense: 5’-GAT CTT GAT CTT CAT GGT GCT AGG-3’
CGRP	Sense: 5′-AAG TTC TCC CCT TTC CTG GT-3′Antisense: 5′-GGT GGG CAC AAA GTT GTC CT-3′
COX-1	Sense: 5′-AGC CCC TCA TTC ACC CAT CAT TT-3′Antisense: 5′-CAG GGA CGC CTG TTC TAC GG- 3′
COX-2	Sense: 5’–ACA ACA TTC CCT TCC TTC-3’Antisense: 5’–CCT TAT TTC CTT TCA CAC C-3’
TNF-α	Sense: 5’-TAC TGA ACT TCG GGG TGA TTG GTC C-3’Antisense: 5’–CAG CCT TGT CCC TTG AAG AGA ACC-3’

Footnotes: CGRP—calcitonin gene-related peptide; COX—cyclooxygenase, TNF—tumor necrosis factor

PCR products were separated by electrophoresis in 2% agarose gel containing 0.5 μg/mL ethidium bromide and then visualized under UV light as described previously [25].

Gastric mucosa of intact rats and those treated with vehicle (saline) and NaHS applied i.g. in different doses with or without PAG was evaluated for the expression of COX-1, COX-2, TNF-α and CGRP mRNAs and the signal intensity was analyzed by densitometry (Gel-Pro Analyzer, Fotodyne Incorporated, Hartland, WI, USA) as described before [20, 25].

### Assessment of COX-1, COX-2, PGE_2_ and TNF-α protein concentration

The concentration of COX-1, COX-2 and PGE_2_ in the gastric mucosa and TNF-α in plasma were determined using direct rat ELISA kits (MyBioSource, San Diego, USA) according to the procedures recommended by a manufacturer. Briefly, tissue homogenates of gastric mucosa were rinsed in ice-cold PBS and homogenized by two freeze-thaw cycles. Homogenates were centrifuged for 15 minutes at 1500×g (or 5000 rpm) and supernatant was stored at −20°C for further analysis. To assess COX-1, COX-2 and PGE_2_ protein concentrations in gastric mucosa homogenates or TNF-α in plasma, 50 μl of each sample and 100 μl of HRP-conjugate reagent was added to the each testing sample well. The plate was incubated for 1 h at 37°C and washed 4 times with wash buffer. Subsequently, Chromogen Solution A (50 μl) and Chromogen Solution B (50 μl) were added to each well and then the microplate was incubated for 15 minutes at 37°C. Then the reaction was stopped with stopping solution and the optical density was read at 450 nm using microplate reader (Biotek Instruments, ELx808, VT, USA).

### Statistical analysis

Results are expressed as mean ± SEM. Statistical comparisons of two groups were performed with Student’s T-test, where appropriate. Comparison involving more than two groups was performed by ANOVA with Tukey *post-hoc* test. A difference with p <0.05 was considered statistically significant. Results are mean ± S.E.M of 6–8 rats per each experimental group.

## Results

### Effect of graded doses of NaHS or L-cysteine on WRS-induced gastric lesions and changes in the GBF

**Fig 1:**
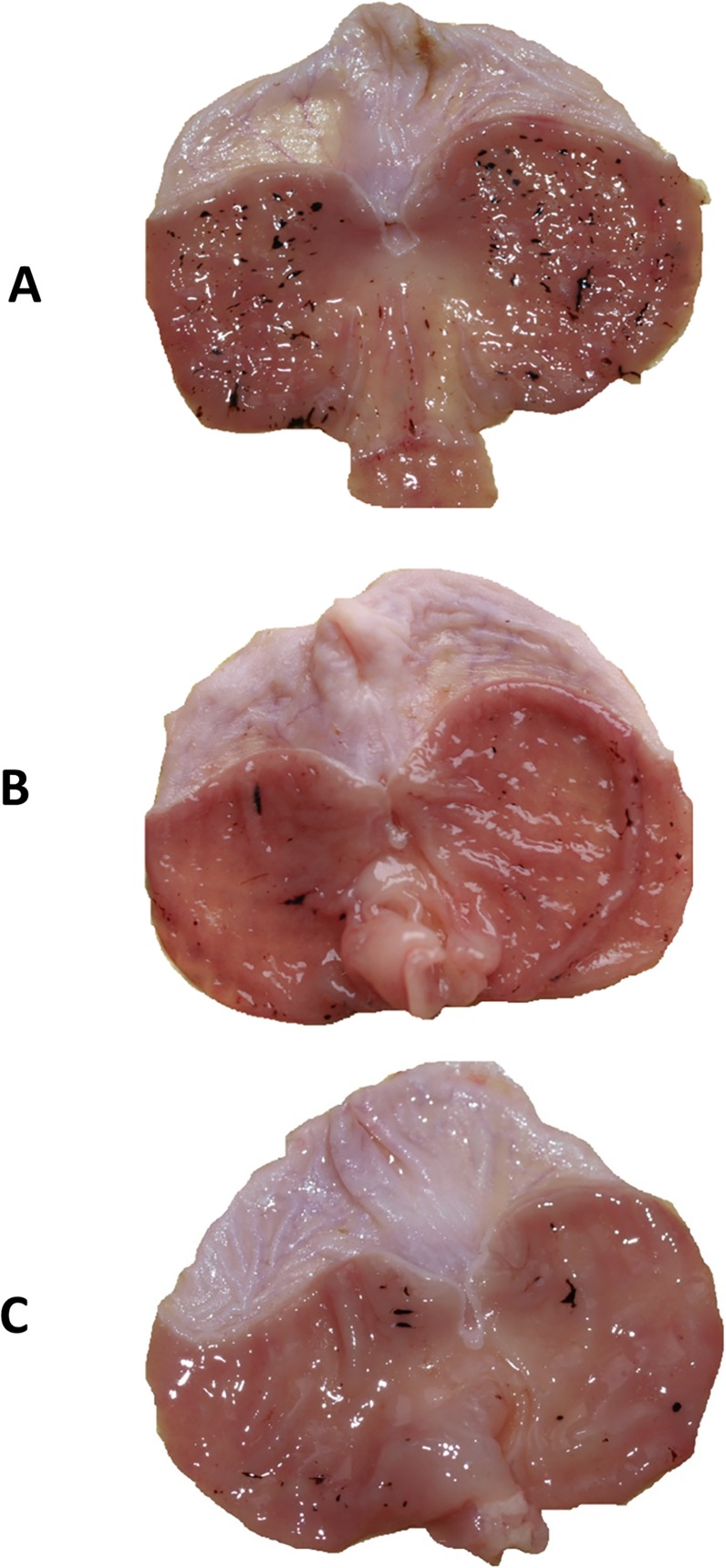
(A, B, C): Macroscopic appearance of rat gastric mucosa of animals exposed to 3.5 h of water immersion and restraint stress (WRS). Rats were pretreated with vehicle (A), NaHS (5 mg/kg i.g.) (B) or L-cysteine (10 mg/kg i.g.) (C). Note, numerous gastric hemorrhagic erosions in gastric mucosa pretreated with vehicle (saline) (Panel A) and significant reduction in the number of gastric lesions in gastric mucosa pretreated with NaHS (Panel B) or L-cysteine (Panel C).

The representative photomicrograph showing mucosal hemorrhagic lesions induced by WRS is presented in Fig. 1A. The exposure of rat to 3.5 h of WRS caused multiple hemorrhagic erosions mainly in the fundic part of the oxyntic mucosa. Pretreatment with NaHS (5 mg/kg i.g.) and L-cysteine (10 mg/kg i.g.) reduced the macroscopic lesions in the gastric mucosa induced by WRS and only a few gastric lesions were observed (Fig. 1B, 1C).

**Fig 2:**
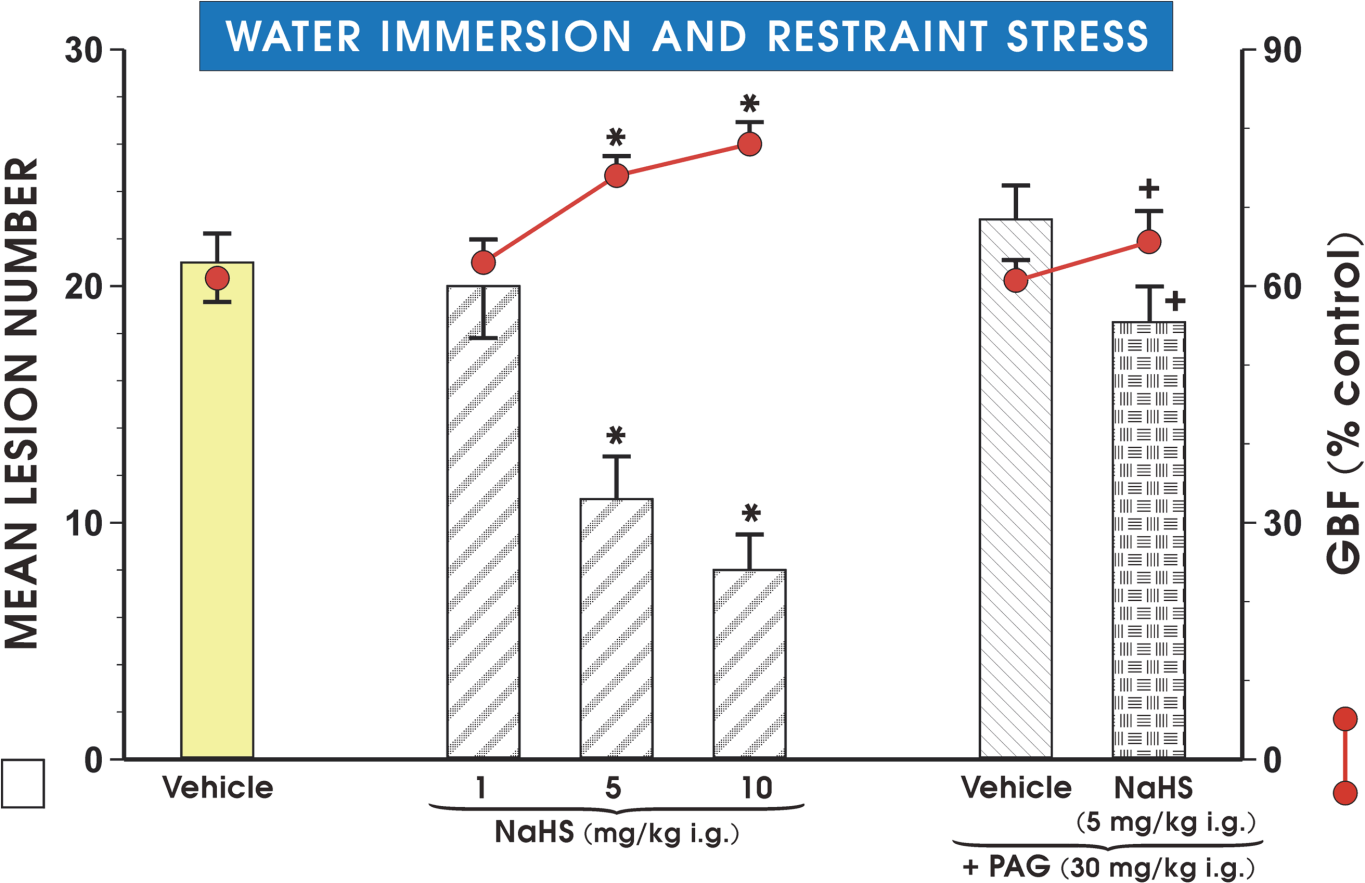
Mean lesion number and the GBF in gastric mucosa of rats pretreated with NaHS. Mean lesion number and the GBF in gastric mucosa of rats exposed to 3.5 h of WRS and pretreated with various doses of NaHS with or without combination with D,L- propargylglycine (PAG, 30 mg/kg i.g.). Control group (Vehicle) was pretreated with saline. Intragastric (i.g.) administration of sodium hydrosulfide (NaHS) in three different doses was ascribed to appropriate groups. One group received NaHS (5 mg/kg i.g.) with and without combination with PAG. Results are mean ±S.E.M of seven rats per each group. Significant change (p<0.05) as compared with the respective values in control group was indicated by asterisk. Cross was used to indicate significant change (p<0.05) comparing to the values obtained in PAG (30 mg/kg i.g.) treated group.

**Fig 3:**
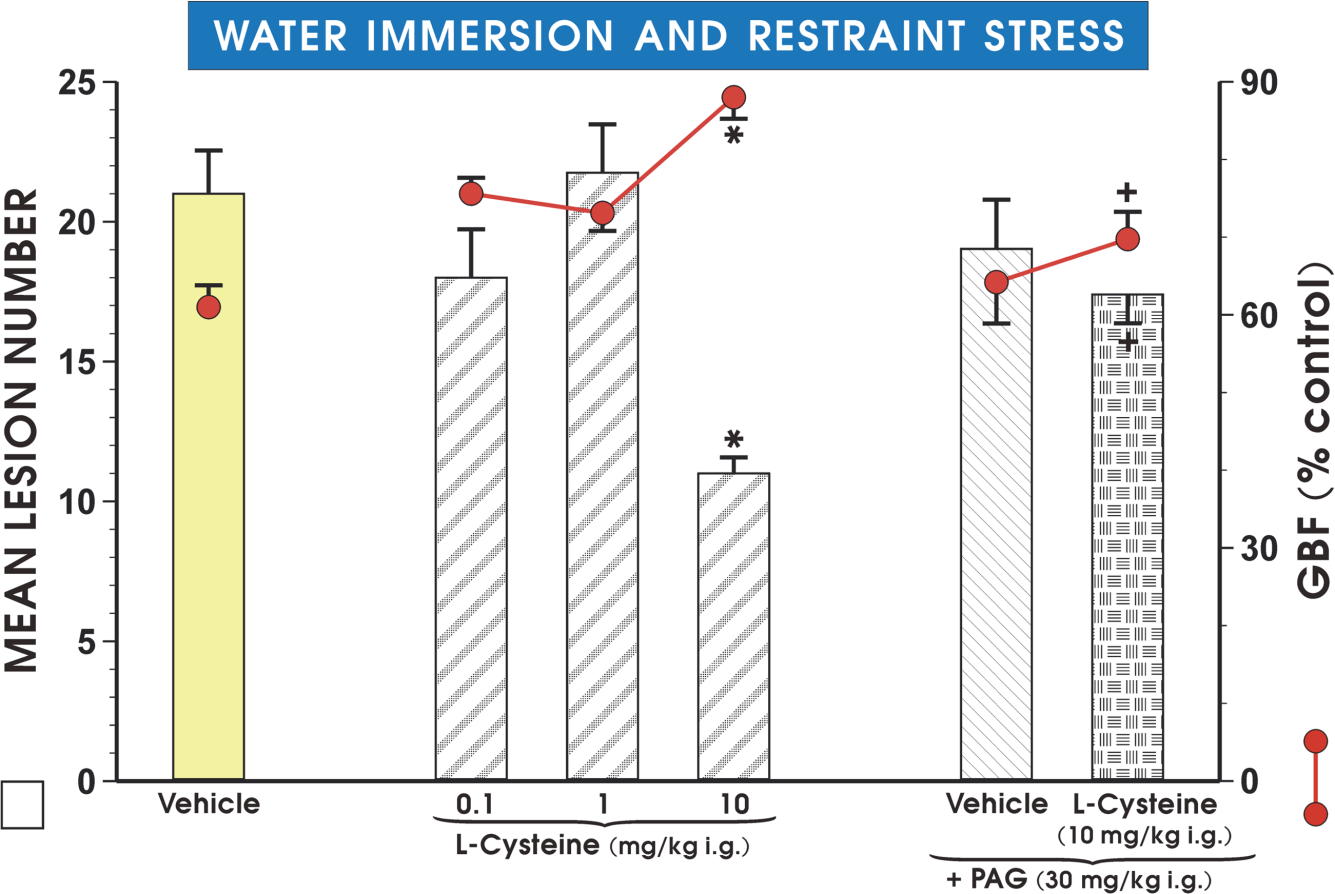
Mean lesion number and the GBF in rats pretreated with L-cysteine. Rats received pretreatment with L-cysteine with or without the combination with PAG, before exposure to 3.5 h of WRS. Control group (Vehicle) was pretreated with placebo (saline). Intragastric (i.g.) administration of L-cysteine in three different doses was ascribed to appropriate groups. One group received D,L- propargylglycine (PAG, 30 mg/kg i.g.) with or without the combination with L-cysteine (10 mg/kg i.g.). Results are mean ±S.E.M of eight rats per each group. Significant change (p<0.05) as compared with the respective values in control group was indicated by asterisk. Cross indicates a significant change (p<0.05) comparing to the values obtained with PAG alone.

As shown in Fig. 2, pretreatment with NaHS dose-dependently attenuated WRS- induced gastric lesions, while producing a significant increase in the GBF with a dose of NaHS which inhibited WRS lesions by 50% being approximately 5 mg/kg. PAG (30 mg/kg i.g.) failed itself to affect the WRS-induced gastric lesions (Fig. 2). A NaHS-induced decrease in the lesion number and an accompanying rise in the GBF were completely reversed by PAG combined with NaHS. Likewise, L-cysteine administered in graded doses ranging from 0.1 mg/kg to 10 mg/kg (Fig. 3), dose-dependently reduced WRS-induced lesions and raised the GBF; the dose which inhibited number of these lesions by 50%, was approximately 10 mg/kg. PAG alone administered in a dose of 30 mg/kg i.p. had no significant influence on WRS lesions, but reversed the decrease in lesion number and an increase in the GBF induced by L-cysteine (10 mg/kg i.g.).

### Assessment of H_2_S production determined by CSE/CBS and 3-MST activity in gastric mucosa of rats pretreated with vehicle or NaHS alone with or without the combination with PAG

**Fig 4:**
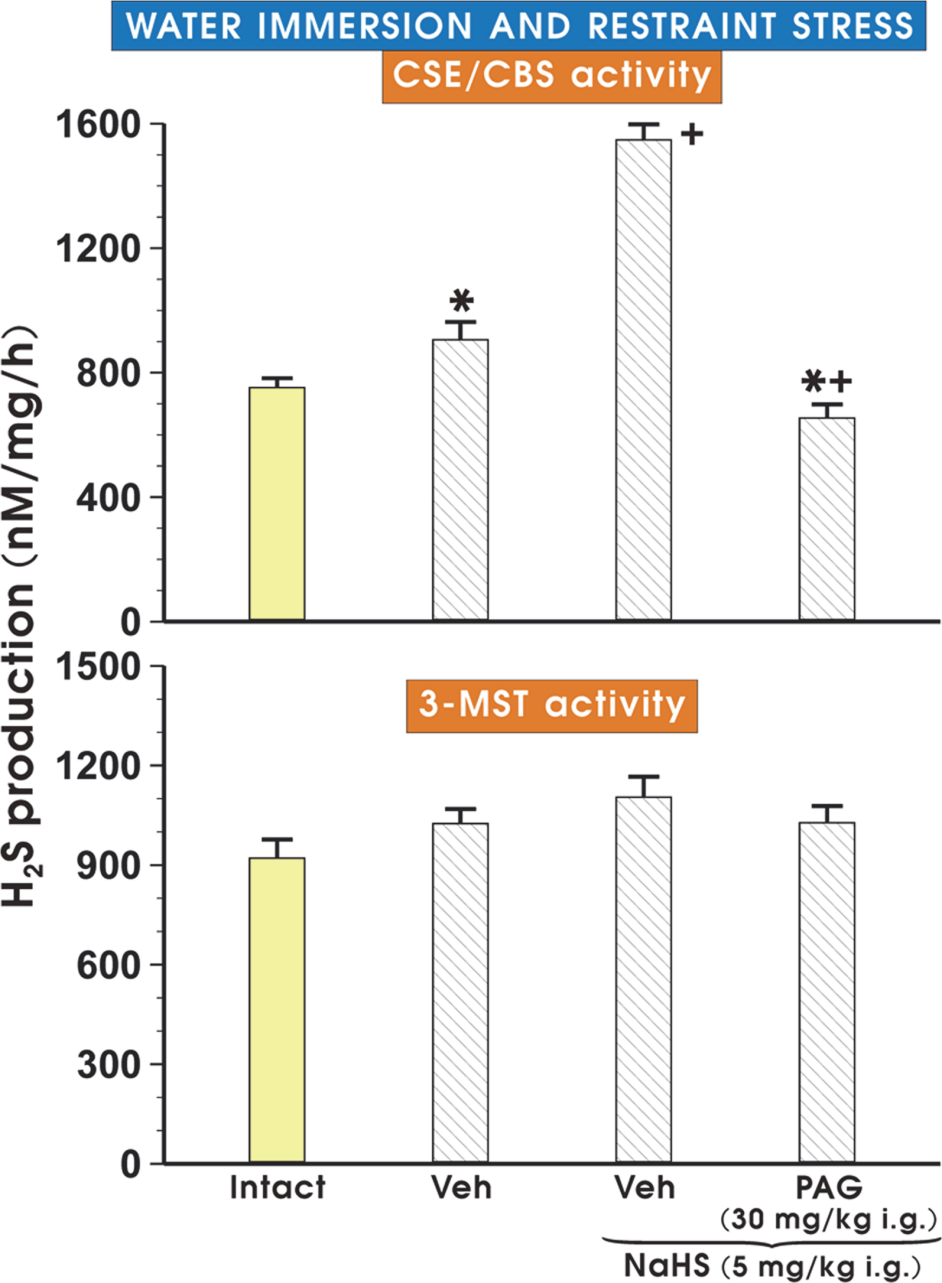
H_2_S production in gastric mucosa determined by CSE/CBS/3-MST activity. The effect of vehicle or NaHS (5 mg/kg i.g.) with or without the combination with PAG (30 mg/kg i.g.) on the concentration of H_2_S determined as the activity of CSE/CBS (upper panel) or the activity of 3-MST (lower panel) in gastric mucosa of rats exposed to WRS. Results are mean ± S.E.M of six rats per each group. Significant change (p<0.05) as compared with the respective values in intact rats was indicated by asterisk. Cross indicates significant change (p<0.05) comparing to the values obtained in vehicle (control) group. Cross and asterisk indicate a significant change (p<0.05) comparing to the values obtained in group treated with NaHS (5 mg/kg i.g.) only.

Fig. 4 (upper and lower panels) shows the effect of vehicle or NaHS (5 mg/kg i.g.) with or without the combination with PAG (30 mg/kg i.g.) on the concentration of H_2_S as determined by activity of CSE/CBS or 3-MST in gastric mucosa of rats exposed to WRS. The concentration of H_2_S produced in gastric mucosa determined by the enzymatic activity of CSE/CBS was significantly increased (p<0.05) in vehicle-control rats exposed to WRS as compared with the concentration of H_2_S in intact gastric mucosa. This increase in the H_2_S concentration in rats exposed to WRS was further significantly elevated (p<0.05) in rats pretreated with NaHS comparing to vehicle-pretreated rats (Fig. 4, upper panel). This increase in the H_2_S concentration was significantly inhibited (p<0.02) when rats received the combination of PAG and NaHS (Fig. 4, upper panel). As shown in the Fig. 4 (lower panel), the concentration of H_2_S determined by 3-MST activity was not significantly altered in rats treated with either vehicle or NaHS with or without the combination with PAG as compared with the concentration of H_2_S measured in intact gastric mucosa.

### The effect of COX-1 and COX-2 inhibitors on the lesion number and changes in the GBF in rats treated with NaHS and L-cysteine

**Fig 5:**
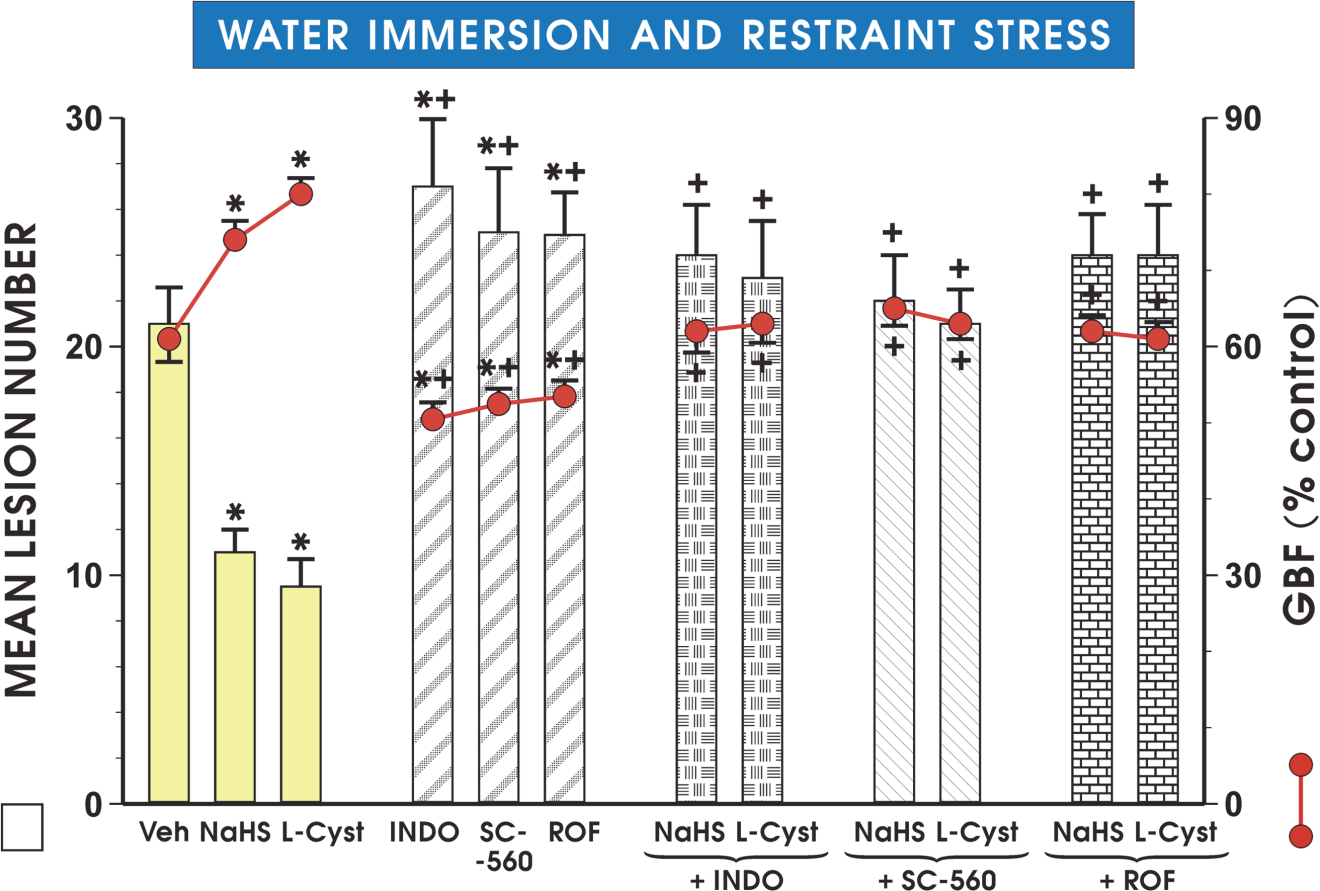
Mean lesion number and GBF in gastric mucosa of rats pretreated with NaHS or L-cysteine combined with COX-1 and COX-2 inhibitors. Before exposure to 3.5 h of WRS, NaHS and L-cysteine (L-cyst) were administered i.g. in doses of 5 mg/kg and 10 mg/kg, respectively. Indomethacin (INDO), SC-560 and rofecoxib (ROF) were administered to appropriate groups with and without combination with NaHS and L-cysteine. Results are mean ± S.E.M of seven rats per each group. Significant change (p<0.05) as compared with the respective values in control group was indicated by asterisk. Asterisk and cross indicate a significant (p<0.05) increase in mean lesion number and decrease in GBF as compared to respective values obtained in vehicle-control animals. Cross indicates significant change (p<0.05) comparing to the values obtained in group treated with NaHS (5 mg/kg i.g.) and L-cysteine (10 mg/kg i.g.) without any combination with COX-1 and COX-2 inhibitors.

As shown in Fig. 5, pretreatment with NaHS (5 mg/kg i.g.) and L-cysteine (10 mg/kg i.g.) resulted in a similar reduction in the lesion number and an increase in the GBF as presented in Figs. 2 and 3. Indomethacin, SC-560 or rofecoxib when administered prior to the onset of WRS, significantly increased the mean lesion number and also significantly decreased the GBF (p<0.05) compared with the pretreated vehicle-control (Fig. 5). The reduction of WRS lesion number and accompanying increase in the GBF induced by NaHS (5 mg/kg i.g.) and L-cysteine (10 mg/kg i.g.) were completely reversed by concurrent treatment with indomethacin, SC-560 and rofecoxib (p<0.05) (Fig. 5).

### The effect of capsaicin-induced deactivation of sensory nerves and blockade of VR-1 receptors by capsazepine on NaHS- and L-cysteine-induced protection

**Fig 6:**
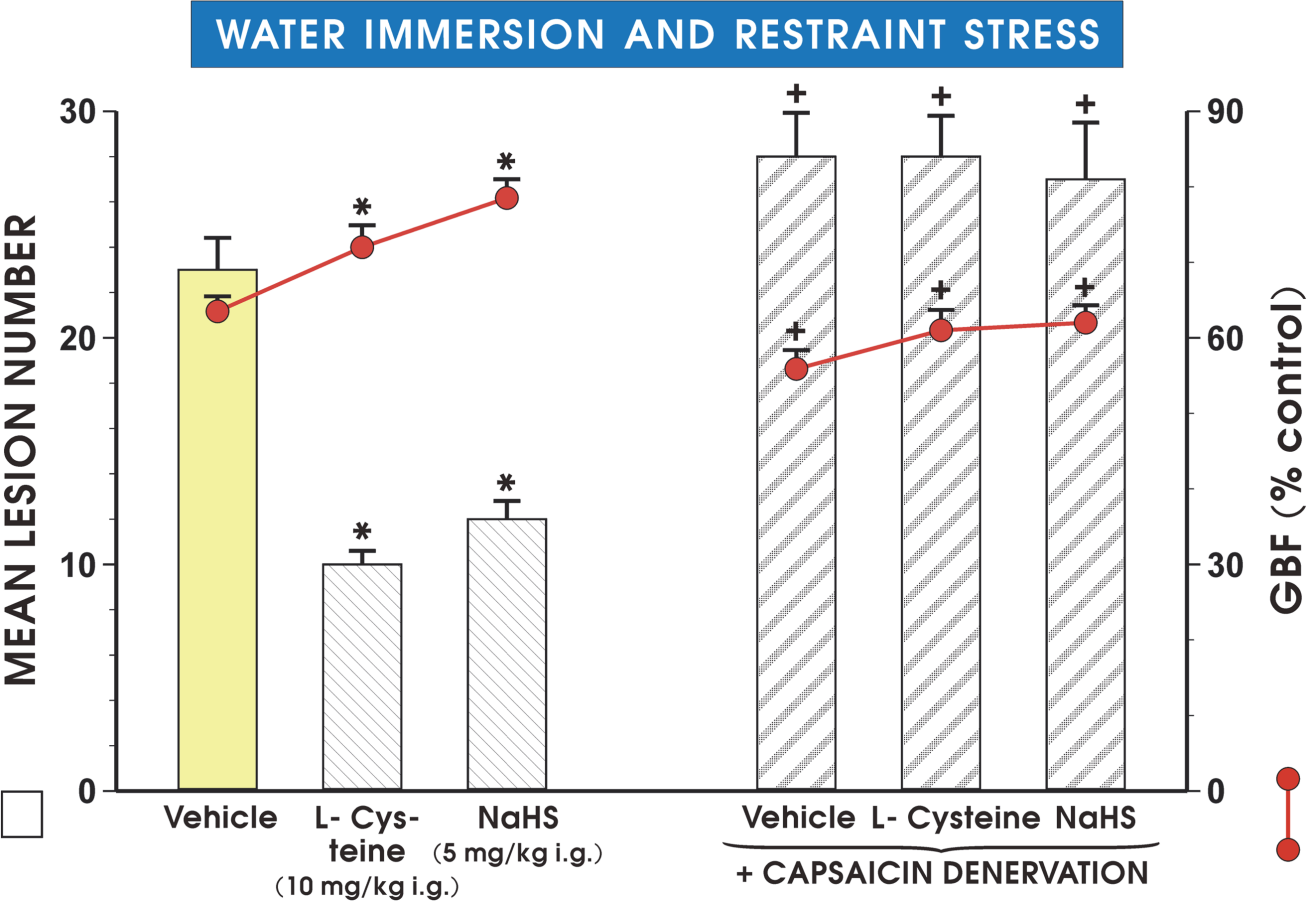
Effect of capsaicin denervation on mean lesion number and GBF in rats exposed to WRS. Rats with intact and capsaicin-denervated sensory neurons were pretreated with vehicle (saline), NaHS (5 mg/kg i.g.) or L-cysteine (10 mg/kg i.g.) and exposed 30 min later to 3.5 h of WRS. Results are mean ±S.E.M of six rats per each group. Significant change (p<0.05) as compared with the respective values in vehicle-control group was indicated by asterisk. Cross indicates significant change (p<0.05) comparing to the values obtained in group treated with saline, NaHS and L-cysteine without denervation.

**Fig 7:**
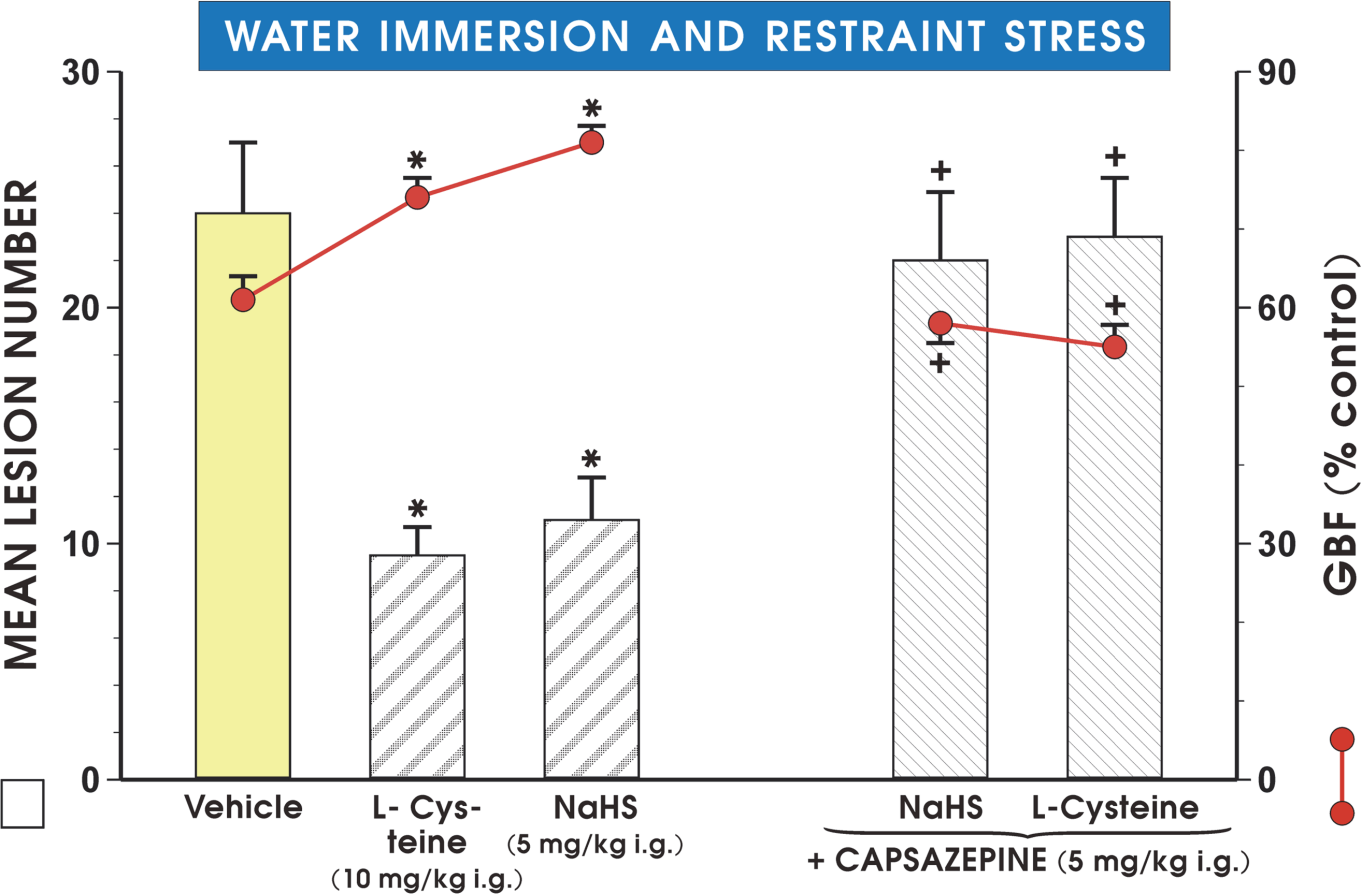
Mean lesion number and the GBF in rats pretreated with capsazepine. WRS-exposed rats were initially administered with vehicle (saline), NaHS (5 mg/kg i.g.) or L-cysteine (10 mg/kg i.g.) with or without combination with capsazepine (5 mg/kg i.g.). Results are mean ±S.E.M of seven rats per each group. Significant change (p<0.05) as compared with the respective values in control group was indicated by asterisk. Cross indicates significant change (p<0.05) comparing to the values obtained in group treated with NaHS and L-cysteine without pretreatment with capsazepine.

As shown in Fig. 6 pretreatment with NaHS or L-cysteine applied i.g. in a dose of 5 mg/kg or 10 mg/kg, respectively, significantly reduced the mean lesion number of WRS-induced gastric lesions and caused the similar significant increase in the GBF as presented in Fig. 5. This attenuation in WRS lesion number and an accompanying increase in the GBF evoked by NaHS and L-cysteine were significantly reversed in capsaicin-denervated animals (p<0.05) (Fig. 6). Fig. 7 shows the effect of pretreatment with capsazepine (5 mg/kg i.g.) alone or that combined with L-cysteine or NaHS on WRS-induced gastric damage and the alterations in the GBF. NaHS and L-cysteine administered alone resulted in similar reduction of WRS-induced gastric lesions and an increase in the GBF as shown in Figs. 5 and 6 and these protective effects of NaHS or L-cysteine were completely lost in the presence of capsazepine (Fig. 7).

### The effect of NaHS administered alone or combined with PGE_2_ synthetic analog on WRS ulcerogenesis and plasma TNF-α levels in rats with intact sensory nerves and those with functional ablation of sensory nerves by capsaicin

**Fig 8:**
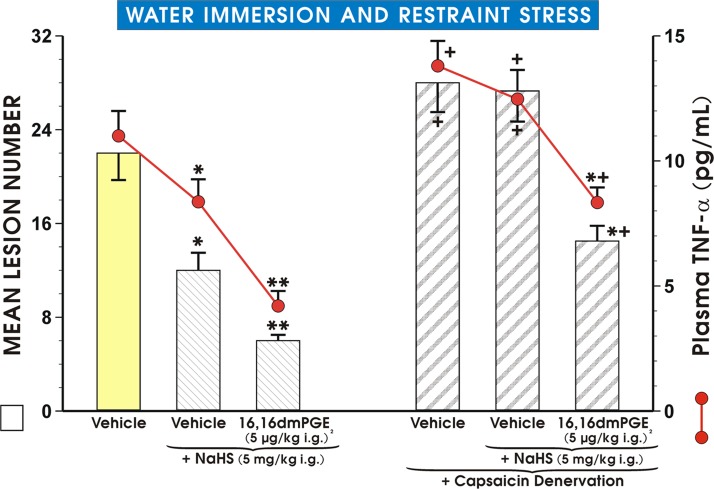
Effect of pretreatment with NaHS alone and in combination with PGE_2_ on mean lesion number and TNF-α plasma levels in WRS rats with or without capsaicin denervation. Rats with intact and capsaicin-denervated sensory neurons were pretreated with vehicle (saline), NaHS (5 mg/kg i.g.) or NaHS combined with 16,16 dmPGE_2_ (5 μg/kg i.g.) and exposed 30 min later to 3.5 h of WRS. Results are mean ± S.E.M of six rats per each group. Significant change (p<0.05) as compared with the respective values in vehicle-control group was denoted with asterisk. Double asterisks indicate a significant change (p<0.05) as compared with NaHS (5 mg/kg i.g.)-pretreated group without capsaicin-denervation. Cross indicates a significant change (p<0.05) comparing to the values obtained in respective control groups treated with saline and NaHS without capsaicin-denervation. Cross and asterisk represent significant change (p<0.05) as compared with vehicle- or NaHS-pretreated group without 16,16 dmPGE_2_ in rats with capsaicin denervation.

As shown in Fig. 8, a similar degree of protection as reflected by the decrease in the mean lesion number was observed in NaHS-pretreated rats exposed to WRS as presented in Figs. 6 and 7 NaHS given in a dose of 5 mg/kg i.g. significantly reduced the plasma concentration of TNF-α (p<0.05) as compared with the value of TNF-α detected in vehicle-pretreated WRS-exposed animals. The combined administration of synthetic analog of PGE_2_ and NaHS further significantly diminished the mean lesion number and plasma TNF-α levels (both at p<0.05) as compared with the respective values of lesion number and plasma concentration of TNF-α in WRS rats pretreated with NaHS alone (Fig. 8). Both, the mean lesion number and plasma TNF-α levels were significantly increased (p<0.05) in rats with capsaicin denervation over the values in vehicle-pretreated rats with intact sensory nerves (Fig. 8). The NaHS-induced reduction in the mean lesion number and the accompanying significant fall in the plasma concentration of TNF-α were significantly decreased (p<0.05) in rats with capsaicin denervation as compared with the respective groups of animals with intact sensory nerves (Fig. 8). When 16,16 dmPGE_2_ was combined with NaHS in rats with sensory denervation, the significant reduction of mean lesion number and plasma TNF-α levels (p<0.05) were recorded, though these reductions failed to reach the similar level as observed in rats with intact sensory nerves treated concomitantly with NaHS and synthetic PGE_2_ analog (Fig. 8).

### The effect of vehicle and NaHS with and without combination with PAG on the mRNA expression of COX-1, COX-2, TNF-α and CGRP

**Fig 9:**
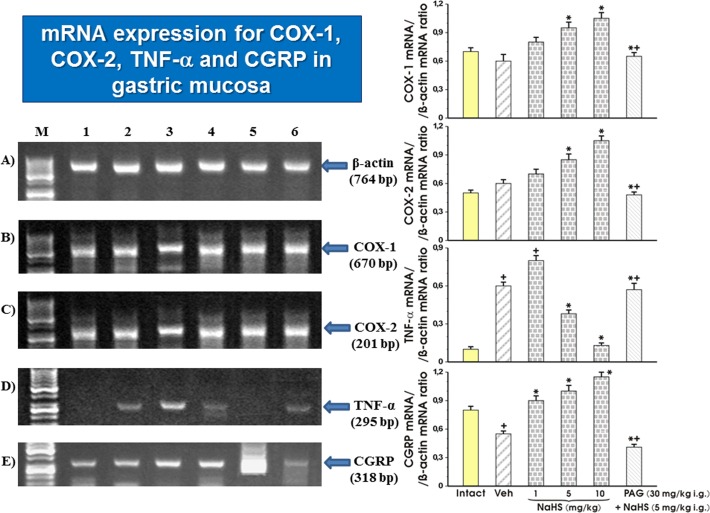
A, B, C, D, E: Expression of mRNA for COX-1, COX-2, TNF-α and CGRP in gastric mucosa. The expression of mRNA for COX-1, COX-2, TNF-α and CGRP was determined in intact rats and those pretreated with vehicle (saline) or H_2_S-donor sodium hydrosulfide (NaHS) with and without combination with D, L- propargylglycine (PAG) and exposed 30 min later to 3.5 h of WRS (panel A). Semi-quantitative densitometry analysis of mRNA expression of COX-1, COX-2, TNF-α, CGRP transcripts normalized to β-actin mRNA expression (panel B). Results are mean ± S.E.M of four determinations. Significant change (p<0.05) as compared with the respective values in vehicle-control gastric mucosa was indicated by asterisk. Cross indicates a significant change (p<0.05) as compared to the value obtained in intact gastric mucosa. Asterisk and cross indicate a significant change (p<0.05) as compared to the values obtained in NaHS (5 mg/kg i.g.) alone pretreated group.

Fig. 9 A-E shows the effect of pretreatment with vehicle, NaHS applied alone in graded doses ranging from 1 mg/kg up to 10 mg/kg and NaHS (5 mg/kg i.g.) combined with PAG on the expression of mRNA for β-actin, COX-1, COX-2, TNF-α and CGRP in gastric mucosa of intact rats and those exposed to WRS. The strong signal for expression of COX-1 mRNA was recorded in intact mucosa and in those pretreated with vehicle. In rats pretreated with NaHS applied in the doses of 5 mg/kg and 10 mg/kg, the signal intensity was significantly enhanced as compared to the intact gastric mucosa or those pretreated with vehicle (Fig. 9, left panel). This increase in signal intensity achieved in rats given NaHS in a dose of 5 mg/kg was significantly inhibited in group of rats treated with NaHS in the presence of PAG. Ratio of COX-1 mRNA over β-actin mRNA confirmed that the COX-1 mRNA was upregulated by NaHS and that this effect of NaHS (5 mg/kg i.g.) was significantly inhibited by PAG (p<0.05) (Fig. 9, right panel). The expression of COX-2 mRNA was detected as a strong signal in vehicle-pretreated gastric mucosa of WRS animals and this effect was further enhanced by NaHS applied in graded concentrations ranging from 1 mg/kg up to 10 mg/kg. The signal for increased expression of COX-2 by NaHS administered at a dose of 5 mg/kg was significantly inhibited by PAG. The ratio of COX-2 mRNA over β-actin mRNA confirmed that the expression of COX-2 mRNA was significantly increased in the vehicle-control gastric mucosa and this effect was further significantly elevated in rats pretreated with NaHS administered in higher doses of 5 mg/kg and 10 mg/kg (p<0.05) (Fig. 9, right panel). The ratio of COX-2 mRNA over β-actin mRNA confirmed that the expression of COX-2 mRNA was significantly inhibited (p<0.05) in rats treated with combination of NaHS and PAG (Fig. 9, right panel). As shown in Fig. 9 (left panel), the signal of expression of TNF-α mRNA was negligible in intact gastric mucosa but it appeared as detectable signal in vehicle-pretreated gastric mucosa and this effect persisted with almost the same signal intensity in gastric mucosa of rat administered with the dose of 1 mg/kg of NaHS. When higher doses of NaHS, 5 mg/kg and 10 mg/kg were applied, the signal intensity of TNF-α mRNA was gradually inhibited. This inhibition of TNF-α expression by NaHS applied at the dose of 5 mg/kg was reversed in part by PAG combined with NaHS. The semiquantitive ratio of TNF-α over β-actin mRNA confirmed that NaHS significantly inhibited the expression of TNF-α (p<0.05), and that concurrent treatment with PAG abolished in part, the inhibitory effect of NaHS on TNF-α mRNA expression (Fig. 9, right panel). The CGRP mRNA was expressed in intact gastric mucosa as a strong signal. In contrast, the vehicle-pretreated gastric mucosa showed weaker signal intensity for CGRP mRNA. In rats pretreated with NaHS applied in graded concentrations ranging from 1 mg/kg up to 10 mg/kg, strong signals for CGRP mRNA were observed (Fig. 9E, left panel). The increase in the signal intensity of CGRP mRNA evoked by NaHS was dramatically reduced by PAG administered in combination with NaHS. The ratio of CGRP mRNA over β-actin mRNA confirmed that NaHS dose-dependently increased the expression of mRNA for CGRP (p<0.05) and this effect of NaHS applied at a dose of 5 mg/kg was significantly diminished by concurrent treatment with PAG (p<0.05) (Fig. 9, right panel).

### The effect of pretreatment with vehicle and NaHS with and without PAG administration or capsaicin-induced sensory nerves inhibition on gastric mucosal protein levels of COX-1, COX-2 and PGE_2_ biosynthesis

**Fig 10:**
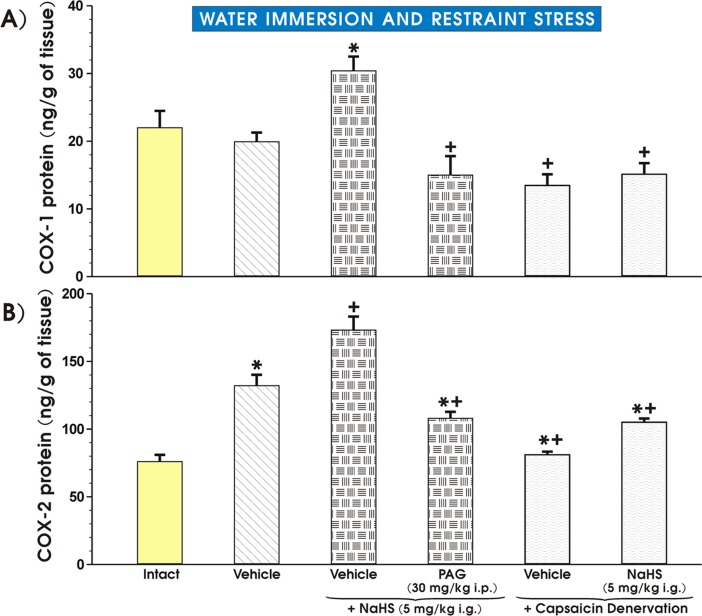
A, B: The effect of pretreatment with vehicle and NaHS with and without PAG administration or capsaicin-induced sensory nerves inhibition on gastric mucosal protein levels of COX-1 and COX-2. Rats with and without capsaicin-denervated sensory neurons were pretreated with vehicle (saline), NaHS (5 mg/kg i.g.) combined or not with PAG (30 mg/kg i.g.) and exposed 30 min later to 3.5 h of WRS. Intact group of rats did not undergo any procedures. Results are mean ±S.E.M of six rats per each group. Asterisk indicates significant change (p<0.05) as compared with the respective values in vehicle-control group (panel A) or with Intact rats (panel B). Cross indicates significant change (p<0.05) as compared with NaHS (5 mg/kg i.g.) pretreated group without capsaicin-denervation (panel A) or in vehicle-control group without capsaicin-denervation (panel B). Cross and asterisk represent significant change (p<0.05) as compared with NaHS pretreated group without capsaicin denervation.

As shown in Fig. 10A, the gastric mucosal COX-1 protein concentration tended to decrease in the gastric mucosa of vehicle-pretreated animals exposed to WRS comparing to intact gastric mucosa but this effect failed to reach statistical significance. In contrast, the COX-1 protein concentration significantly increased (p<0.02) in rats pretreated with NaHS and this increase was significantly attenuated by concurrent administration of PAG (p<0.05) (Fig. 10 A). The COX-1 protein content was significantly decreased in capsaicin denervated animals pretreated with vehicle and exposed to WRS as compared to those with intact sensory nerves pretreated with vehicle and further exposed to WRS (Fig. 10 A). The increase in COX-1 protein concentration achieved in rats with intact sensory nerves pretreated with NaHS was significantly decreased (p<0.05) in those pretreated with NaHS with capsaicin denervation (Fig. 10 A). Fig. 10 B shows the effect of pretreatment with vehicle or NaHS with or without combination with PAG (30 mg/kg i.p.) or the inhibition of sensory nerves on COX-2 gastric mucosal protein content in rats with intact sensory nerves or in those with capsaicin denervation. The significant increase in COX-2 protein content (p<0.05) was observed in vehicle-pretreated rats exposed to WRS as compared with the COX-2 concentration in the intact gastric mucosa (Fig. 10 B). In rats pretreated with NaHS before onset of WRS, the significant rise in the COX-2 protein content (p<0.02) was observed over that recorded in vehicle-treated gastric mucosa exposed to WRS and this increase in COX-2 protein content was significantly decreased (p<0.05) in rats treated with combination of PAG and NaHS (Fig. 10 B). The protein COX-2 content was significantly decreased (p<0.05) in capsaicin denervated rats pretreated with vehicle or NaHS as compared with the respective values of COX-2 protein in rats with intact sensory nerves pretreated with vehicle or NaHS (Fig. 10 B).

Table 2 presents the data with the gastric mucosal PGE_2_ content in intact rats and in those pretreated with vehicle (saline) or NaHS (5 mg/kg i.g.) with or without combination of PAG (30 mg/kg i.p.) or capsaicin sensory denervation. The PGE_2_ generation was insignificantly altered in vehicle-pretreated rats exposed to WRS as compared with intact gastric mucosa. In contrast, the gastric mucosal PGE_2_ generation was significantly increased (p<0.05) in animals pretreated with NaHS and this effect was significantly decreased in rats administered with the combination of PAG and NaHS (Table 2). The generation of PGE_2_ was significantly inhibited in gastric mucosa of capsaicin denervated rats (Table 2). The increase in the PGE_2_ content evoked by NaHS was significantly (p<0,05) diminished in rats with functional ablation of sensory nerves induced by capsaicin.

**Table 2:** Effect of pretreatment with vehicle (saline) or NaHS (5 mg/kg i.g.) applied alone or combined with PAG (30 mg/kg i.p.) or the inhibition of sensory nerves by capsaicin on the PGE_2_ concentration in the gastric mucosa of intact rats or those exposed to 3.5 hrs of WRS.

EXPERIMENTAL GROUP	GASTRIC MUCOSAL PGE_2_ CONTENT (ng/mg of tissue weight)
Intact	33 ± 3.5
Vehicle	42 ± 4.3
NaHS (5 mg/kg i.g.)	56 ± 4.7*
PAG (30 mg/kg i.g.) + NaHS	28 ± 2.6^+^
Vehicle + capsaicin denervation	24 ± 2.8^+^
NaHS + capsaicin denervation	27 ± 3.1^+^

Results are mean ± S.E.M of eight rats per each group.

* Asterisk denotes a significant change (p<0.05) as compared with the respective values in vehicle (control) group.

^+^ Cross indicates a significant change (p<0.05) as compared to the values obtained in NaHS alone pretreated group.

## Discussion

Current evidence indicates that H_2_S is a gaseous transmitter involved in the control of vascular tone and GI motility, however, its mechanism of gastroprotection has not been thoroughly studied [12]. In particular, little is known whether donors of H_2_S could exert the protective action against the formation of acute gastric lesions such as those induced by stress and whether the activity of the afferent sensory nerves and endogenous PGs could be involved in this protection.

We selected a stress animal model of WRS to determine the mechanism of action of H_2_S on the gastric mucosa, because of clinical relevance of stress as a potent risk factor of micro bleeding erosions and even peptic ulcers in humans [26]. We reported that the fall in gastric microcirculation and the formation of bleeding gastric lesions are observed in rats under experimental conditions of stress [22, 27]. This animal model of WRS mimics hemorrhagic erosions that can appear as a consequence of stress bleedings under life threatening conditions in humans [17, 23, 26, 28].

Herein, we demonstrated that pretreatment with L-cysteine, a H_2_S precursor, or NaHS, a H_2_S donor, protects the gastric mucosa against acute mucosal lesions induced by WRS, because the mean lesion number of WRS damage was decreased in rats treated with either H_2_S precursor or H_2_S donor and these protective effects of L-cysteine or NaHS were accompanied by an increase of the GBF. We demonstrated that exposure to WRS increased activity of key enzymatic CSE/CBS pathway resulting in an elevated H_2_S production *in vitro* in gastric mucosa suggesting that this gastric mucosa can compensate for the damaging effect of stress by an increase of H_2_S production to enhance the self-defense mucosal protective mechanism against the damage under stress conditions. This observation is in keeping with previous findings [13, 15] that the induction of an ulcer in the upper or lower gut results in a marked increase in H_2_S synthesis and production, possibly due to the compensatory protective mechanism in the gastrointestinal mucosa predisposed to the injury evoked by various ulcerogenes. Moreover, we found for the first time that NaHS further increased H_2_S production in the gastric mucosa, suggesting the “reciprocal” interaction of H_2_S donor with its endogenous product H_2_S endorsing its protective and hyperemic effects against stress ulcerogenesis. Our study supports the notion that CSE/CBS but not 3-MST pathway is responsible for the H_2_S production in gastric mucosa exposed to WRS because at our experimental conditions, NaHS administered with or without the combination with PAG failed to alter the 3-MST pathway. The role of H_2_S in mucosal protection is supported by our present finding that L-cysteine, the H_2_S precursor, also exerted the protective and hyperemic activity against WRS ulcerogenesis. These observations clearly suggest that H_2_S-induced protection could be due to an increase in the GBF mediated by endogenous H_2_S and vasorelaxatory action of H_2_S donor and its precursor. These results are consistent with the findings of Fiorucci et al. [29], who also reported that H_2_S donors reduced NSAID-induced leukocyte adherence in the gastric microcirculation. Neutrophil adherence induced by NSAIDs has been suggested to act as a primary mechanism for the gastric-damaging effect of these drugs [30].

Interestingly, NaHS can exert either vasodilatation or vasoconstriction depending on the dose employed [10]. In the dose range used in our present study NaHS exhibited vasodilatory properties as documented by dose-dependent increase in GBF that accompanied the protective activity of this compound suggesting that H_2_S is responsible for an increase in the GBF observed in our study. Our data are corroborative with recent report that NaHS administered intraperitoneally prevented cold restraint stress-induced oxidative gastric damage by the mechanism involving the inhibition of gastric acid and attenuation of reactive oxygen metabolites [31]. Lou *et al*. [32] demonstrated that beneficial effect of H_2_S may depend upon hypothermia caused by H_2_S that reduced the concentration of the lipid peroxidation products in the gastric mucosa exposed to stress, thus attenuating stress ulcerations. Moreover, it was shown that NaHS protected gastric mucosal epithelial cells against H_2_O_2_-induced cell death [33]. H_2_S can inhibit gastric lesions caused by topical damaging agents including non-steroid anti-inflammatory drugs (NSAID), such as diclofenac and naproxen [34, 35]. In relevance to these findings, the new and safer NSAID releasing H_2_S such as H_2_S-releasing diclofenac (ATB-337) and H_2_S-releasing naproxen (ATB-346) were shown to reduce gastric lesions induced by conventional NSAID [34, 35].

We found that CSE inhibitor-PAG, which in the presence of NaHS markedly decreased the H_2_S production as analyzed in this study by CSE/CBS pathway and reversed the NaHS-induced protection against stress-induced gastric lesions and accompanying increase in the GBF. However, in another study [36], the administration of PAG resulted in protection against ethanol injury and this was explained by evident reduction in H_2_S concentration elevated in response to intragastric application of ethanol. Moreover, indomethacin inhibited the protective effects of PAG, thus suggesting the involvement of endogenous PGs in gastroprotection exerted by this CSE inhibitor [36]. This apparent discrepancy in results with PAG between their results [36] and our present study could be attributed to differences in mechanism of damage and protection against chemical and non-topical damage induced by absolute ethanol used in their report [36] and by non-topical ulcerogenesis such as stress in our present study. Moreover, using similar experimental model of stress-induced gastric lesions, Aboubakr *et al*. [31] demonstrated that the inhibition of key enzyme of H_2_S synthesis, CSE with beta-cyano-L-alanine (BCA) exacerbated stress-induced gastric damage.

We clearly demonstrated that endogenous PGs and PG/COX system are involved in the mechanism of NaHS-induced gastroprotection because in rats pretreated with NaHS the upregulation of mRNA expression of COX-1 and COX-2 and the rise in the COX-1 and COX-2 protein concentrations were observed. Furthermore, we provided evidence that the pretreatment with PAG in combination with NaHS not only reversed protective and hyperemic effect of NaHS but also downregulated the expression of mRNA for COX-1 and COX-2 and NaHS induced increase in COX-1 and COX-2 protein contents.

Previous studies in rats and humans demonstrated that PGE_2_ is present in the largest amounts in the gastric mucosa and exerts gastroprotective action within upper GI tract [37, 38]. That is why, we determined whether the co-administration of selective (SC-560, rofecoxib) and non-selective (indomethacin) inhibitors of COX-1 and COX-2, known to suppress the mucosal generation of endogenous PGE_2_ [39], could affect the NaHS and L-cysteine-induced protection and changes in the GBF. We found that the concurrent treatment of COX-1 and COX-2 inhibitors with NaHS and L-cysteine almost completely reversed both protective and hyperemic activities exhibited by this H_2_S donor and the H_2_S precursor. Therefore, we conclude that H_2_S-induced gastroprotection and increase in gastric microcirculation may depend upon the activity of PG/COX system. We observed that this NaHS-induced protection against WRS-induced gastric lesions is accompanied by the rise in gastric mucosal PGE_2_ concentration suggesting that the protective and hyperemic actions of H_2_S can involve the activation of COX/PG system and its endogenous products, PG.

The H_2_S-induced rise in COX-1 and COX-2 protein contents and mucosal PGE_2_ concentration were significantly attenuated in rats with capsaicin denervation suggesting that capsaicin—sensitive afferent neurons and sensory vasoactive neuropeptides such as CGRP may contribute to the beneficial protective effect of NaHS in rat stomach. Indeed, the afferent sensory fibers were originally proposed to play an important role in the mechanism of gastric mucosal integrity and gastroprotection [21]. This is why we studied the effects of NaHS and L-cysteine in animals with capsaicin denervation and in those with blockade of VR-1 receptors with capsazepine. We confirmed that capsaicin denervation itself decreased the GBF and augmented WRS-induced ulcerogenesis. The protective and hyperemic activity of NaHS and L-cysteine were greatly attenuated in rats with capsaicin denervation. Taken together, we propose that the mechanism of H_2_S-induced protection could be due to an increased activity of the afferent sensory nerves and a release of CGRP, which activates VR-1 receptors, thus causing vasodilatation. This is supported by the observation that NaHS and L-cysteine protection was accompanied by an increase of mRNA CGRP expression and this effect was attenuated when PAG combined with NaHS or L-cysteine. In order to look for the relation between PGs, altered activity of sensory nerves and proinflammatory cytokine TNF-α in gastroprotection by H_2_S, we tested the effect of NaHS in the presence or absence of synthetic analog of PGE_2_ against WRS ulcerogenesis in rats with or without capsaicin denervation. We found that NaHS-induced protection was potentiated by co-treatment with PGE_2_ analog, and that this effect of combined administration of NaHS and PGE_2_ persisted, though being significantly reduced, in rats with capsaicin denervation. Moreover, the NaHS protection was accompanied by the fall in the plasma TNF-α level, which was potentiated when exogenous PGE_2_ which was co-administered together with H_2_S donor. In contrast, this decrease in the plasma TNF-α level observed in rats concomitantly treated with the combination of NaHS and PGE_2_ was diminished in those with capsaicin denervation suggesting the link between prostaglandins, neuropeptides released from sensory nerves and the release of proinflammatory cytokines such as TNF-α in gastroprotection elicited by H_2_S. We confirmed our previous observation [16] that afferent sensory nerves ablation induced by application of capsaicin increased WRS-induced gastric lesions and decreased GBF as compared with rats with intact sensory nerves exposed to WRS. Moreover, SC-560 and COX-2 inhibitor, rofecoxib were shown to exert the same aggravatory effects as capsaicin denervation. Interestingly, SC-560 or rofecoxib administered to rats with capsaicin-induced sensory nerves ablation in our previous study [16] even enhanced noxious effect of capsaicin denervation suggesting that the impairment of these two pathways can exert deleterious influence on gastric mucosa exposed to WRS. Indeed, as demonstrated in this study, capsaicin denervation and the selective and non-selective inhibition of COX-1/COX-2 pathways reversed the beneficial effects of H_2_S against WRS-induced gastric damage. Additionally, the afferent sensory nerves ablation by capsaicin decreased COX-1 and COX-2 protein contents and the rise in the mucosal generation of PGE_2_ observed in rats pretreated with NaHS. H_2_S-induced gastroprotection may depend upon activity of two synergistic pathways, namely afferent sensory nerves activity releasing CGRP and endogenous PGs derived from the enhanced expression and activity of COX-1 and COX-2.

Interestingly, the systemic (i.v.) administration of L-cysteine and NaHS attenuated gastric injury induced by ischemia/reperfusion [40]. The mechanism of this systemic action of NaHS and L-cysteine has been attributed to a decrease in plasma levels and downregulation of mRNA expression of proinflammatory cytokines IL-1β or TNF-α. These effects of H_2_S donor and H_2_S precursor were diminished by pretreatment with PAG [40]. Herein, we present an evidence that NaHS administration decreased TNF-α mRNA expression and this effect was reversed when NaHS was combined with PAG. Thus, it is likely that gastroprotection induced by NaHS releasing H_2_S exerts the anti-inflammatory activity that also could be an important component of gastroprotection afforded by this gaseous molecule.

In summary, H_2_S released from its donor, NaHS or that synthesized from L-cysteine, plays an important physiological role in gastric mucosal protection against stress-induced lesions. This protection induced by NaHS or L-cysteine is accompanied by an enhancement in in the gastric microcirculation possibly mediated by a significant local increase in the gastric mucosal production of H_2_S. The mechanism of H_2_S-induced gastroprotection involves activation of endogenous PGs/COX system, the rise in biosynthesis of PGE_2_, and afferent sensory fibers releasing CGRP acting *via* VR-1 receptors and by anti-inflammatory effect resulting in the inhibition of pro-inflammatory cytokines such as TNF-α.
